# Systemic barriers to recruitment of blood donors from African, Caribbean, and Black communities

**DOI:** 10.1002/hem3.70414

**Published:** 2026-06-30

**Authors:** Edeghonghon Olayemi

**Affiliations:** ^1^ Richmond Hospital Vancouver Coastal Health Richmond British Columbia Canada

There have been several advances in the management of sickle cell disease (SCD), such as penicillin prophylaxis in children, hydroxyurea, hemopoietic stem cell transplant, gene therapy, and other novel targeted therapies.^1^, ^2^ Despite these advancements, blood transfusion remains an important treatment modality in SCD.^3^ However, racial/genetic diversity in the blood donor pool has an impact on safe blood provision for patients with inherited blood disorders, such as SCD.^4^ One significant drawback in this respect is the high frequency of alloantibodies in chronically transfused people living with SCD. This high alloantibody prevalence is related to the fact that in high‐income countries (HICs), there is a genetic difference between blood donors, who are mostly of Caucasian origin, and people living with SCD, who are mostly of African, Caribbean, and Black (ACB) origin.^5^ Due to blood group polymorphism, blood types tend to be more common in specific populations; so, it is easier to find a matching blood type to a person with SCD among donors who identify as ACB than those from other parts of the world.^6^ Under‐representation of ACB donors in the donor pool has persisted in most HICs despite efforts targeted at improving their representation. A recent qualitative study from Canada using semi‐structured in‐depth interviews by Haw et al.^7^ was aimed at understanding systemic barriers to donation from the perspectives of ACB community leaders. The following barriers were identified.

i. Mistrusting blood collection agencies (BCAs) as an extension of healthcare systems

The authors found out that due to differences in sociocultural beliefs, some key informants had developed a mistrust in BCAs as they were not certain about what their donated blood would be used for. This mistrust was an extension of their prior unfavorable opinion of the healthcare systems. Key informant P07 was quoted as saying:

I think the more I went into public health, the more I realized the mistrust that there is between the Black community and anything that is scientific and medical, there is that, that we don′t trust these people, so the idea to go and give blood, it′s like, what are they going to do with our blood?

Historical events played a role in the development of this mistrust by the ACB community in the healthcare system, including but not limited to events such as the Tuskegee Syphilis Trial in the United States. Lived experiences of racism and discrimination while seeking healthcare as a person of color also contributed to it.

ii. Navigating differing sociocultural views on blood and donation

The dominant Western view that there is no meaningful link between the donor and donated blood is not universal. Some key informants in this study emphasized the importance, meaning, and sacredness of blood to them and their communities. Thus, they would be reluctant to donate without knowing what would happen to their blood. Others were concerned about cultural judgments they faced from BCA staff whenever they raised questions related to their differing views on blood donation. This was not helped by the fact that the ACB community is generally poorly represented among donor care center staff.

iii. Viewing deferral criteria as exclusionary

Some donor deferral criteria were seen as disproportionately affecting people of ACB origin; these were seen as discriminatory and exclusionary. For example, the current malaria criterion in Canada would exclude virtually everyone who has lived in Africa. This deferral criterion may be seen to align with the identity of Africans living in Canada, and from the point of view of the donor, this is seen as an identity‐based deferral since it would apply to virtually everyone from the continent. There is also the issue of historical facts that have been passed down within the ACB community; for example, people from certain African countries were not allowed to donate because of the risk of transmitting HIV variants. As a result, although this requirement was removed several years ago, people from the community are still reluctant to donate. For similar reasons, a particular key informant (P15), who had been a regular donor, was permanently deferred because she was dating someone born in Africa.

## CONCLUSION

This study by Haw et al. identifies barriers faced by potential ACB blood donors in Canada. It highlights factors which have been described earlier by investigators in other HICs such as Australia,^8^ Europe,^4^ the United States,^9^ and Canada.^10^ Their work draws attention to the unending stereotypes faced by people of ACB origin, such as being labeled as drug seekers when seeking medical assistance as persons living with SCD or women in labor, and harbingers of transfusion‐transmissible diseases when trying to voluntarily donate blood. It is then not surprising when some respondents saw BCAs as an extension of the healthcare system, which they already have little or no confidence in. Thus, while the immediate reason for the high rates of alloimmunization seen in people with SCD who require chronic transfusion may be genetic differences between donors and recipients, the actual reason may be the lost opportunity of recruiting sufficient voluntary donors from the ACB community due to their lack of trust in the system and the erection of unnecessary barriers between the community and the BCAs.

## CONFLICT OF INTEREST STATEMENT

The author declares no conflicts of interest.

## FUNDING

This research received no funding.

## Data Availability

Data sharing is not applicable to this article as no datasets were generated or analyzed during the current study.
